# Protective effects of seaweed supplemented diet on antioxidant and immune responses in European seabass (*Dicentrarchus labrax*) subjected to bacterial infection

**DOI:** 10.1038/s41598-019-52693-6

**Published:** 2019-11-06

**Authors:** Maria J. Peixoto, Renato Ferraz, Leonardo J. Magnoni, Rui Pereira, José F. Gonçalves, Josep Calduch-Giner, Jaume Pérez-Sánchez, Rodrigo O. A. Ozório

**Affiliations:** 1 CIIMAR – Centro Interdisciplinar de Investigação Marinha e Ambiental, Universidade do Porto, Av. General Norton de Matos s/n, 4450-208 Matosinhos, Portugal Portugal; 2 ICBAS – Instituto de Ciências Biomédicas de Abel Salazar, Universidade do Porto, Rua de Jorge Viterbo Ferreira 228, 4050-313 Porto, Portugal; 3 ALGAPLUS, Lda - Travessa Alexandre da Conceição S/N, 3830-196 Ílhavo, Portugal; 4 Nutrigenomics and Fish Growth Endocrinology Group, Institute of Aquaculture Torre de la Sal, IATS-CSIC, 12595 Ribera de Cabanes, Castellón Spain; 5 IIB-INTECH - Instituto de Investigaciones Biotecnológicas - Instituto Tecnológico de Chascomús (CONICET), Chascomús, Argentina

**Keywords:** Bacterial infection, Marine biology

## Abstract

European seabass (*Dicentrarchus labrax*) production is often hampered by bacterial infections such as photobacteriosis caused by *Photobacterium damselae* subsp. piscicida (Phdp). Since diet can impact fish immunity, this work investigated the effect of dietary supplementation of 5% *Gracilaria* sp. aqueous extract (GRA) on seabass antioxidant capacity and resistance against Phdp. After infection, mortality was delayed in fish fed GRA, which also revealed increased lysozyme activity levels, as well as decreased lipid peroxidation, suggesting higher antioxidant capacity than in fish fed a control diet. Dietary GRA induced a down-regulation of hepatic stress-responsive heat shock proteins (*grp-78*, *grp-170*, *grp-94*, *grp-75*), while bacterial infection caused a down-regulation in antioxidant genes (*prdx4* and *mn-sod*). Diet and infection interaction down-regulated the transcription levels of genes associated with oxidative stress response (*prdx5* and *gpx4*) in liver. In head-kidney, GRA led to an up-regulation of genes associated with inflammation (*il34*, *ccr9*, *cd33*) and a down-regulation of genes related to cytokine signalling (*mif*, *il1b*, *defb*, *a2m*, *myd88*). Additionally, bacterial infection up-regulated immunoglobulins production (IgMs) and down-regulated the transcription of the antimicrobial peptide *leap2* in head kidney. Overall, we found that GRA supplementation modulated seabass resistance to Phdp infection.

## Introduction

Aquaculture production involves rearing animals at high densities in enclosed spaces, often resulting in deteriorated water quality, affecting fish health and favouring the proliferation of opportunistic bacteria^1,2^. These conditions lead to immunosuppression and the disruption of antioxidant systems, increasing the susceptibility to infectious agents^3^. A common opportunist is the Gram-negative halophilic bacterium *Photobacterium damselae* subsp. piscicida (Phdp), the causative agent of photobacteriosis. This fish disease is known to induce acute septicaemia in young fish or granulomatous lesions in adults^4^ culminating in high mortality rates and massive economic losses for producers^2^. To address this issue, producers favour preventive techniques^5^, such as strengthening fish immunity through the prophylactic administration of immunostimulants and antioxidant supplements^6^. These cost effective and sustainable methods constitute an alternative to vaccines, maximizing the use of natural components in diets formulation, as they are less likely to interfere with fish homeostasis or disrupt the environment^5,7,8^. Thus, seaweeds containing bioactive molecules with immunostimulant and antioxidant properties are in the spotlight to improve robustness of farmed fish without compromising growth^9,10^.

The polysaccharides of seaweeds have been shown to stimulate non-specific host immunity and to inhibit bacterial activity. These carbohydrates also positively modulate gut health and potentiate fish digestive capacities, hallmarks of a prebiotic categorization^10–12^. Additionally, seaweed *β*-glucans were described to stimulate the immune system through the rapid release of reactive oxygen species (ROS) and signalling proteins^13^. Seaweed phenolic compounds may also wield a scavenging effect^14^, reducing ROS formation in fish tissues. Moreover, red seaweeds such as *Gracilaria* sp. are rich in arachidonic acid, the precursor of the pro-inflammatory mediators prostaglandins, thromboxanes and leukotrienes^15,16^. These chemotactic lipids are key players in phagocytosis and antigen presentation^17^, essential to counteract infection.

The importance of European seabass (*Dicentrarchus labrax*) in aquaculture has instigated numerous studies regarding the effect of dietary changes in fish immunity, including the partial replacement of fish oil and fish meal^18–20^. However, long-term feeding with plant derived ingredients remains controversial due to anti-nutritional factors^21^. For instance, diets with high soybean content, have been associated with the activation of T cell mediated processes such as the up-regulation of interleukins IL-18 and IL-22, as well as the transcription of genetic markers of inflammation, namely tumour necrosis factor (TNF-α) and factor nuclear kappa (NF-kB)^22^. Despite the negative impact of plant proteins in fish health, these studies established that stress responsiveness and susceptibility to infection could be nutritionally modulated^23^. More recently, the supplementation of *Gracilaria gracilis* in *Danio rerio* diets resulted in increased immune and antioxidant activities^24^, yet little is known about the mechanisms by which functional foods modulate fish metabolism and immunity, both locally at infection sites and systemically^25^. Therefore, it is imperative for aquaculture to understand how ingredients derived from marine sources, such as seaweeds, can be used in aquafeeds to improve fish immunity.

The present work evaluated the effect of dietary supplementation with 5% *Gracilaria* sp. aqueous extract in seabass when infected with *Photobacterium damselae* subsp. *piscicida*. Specifically, we aimed to determine how *Gracilaria* sp. supplementation affected seabass survival rates, plasma bioindicators levels, immune and antioxidant parameters, as well as immune and antioxidant genes transcription in response to infection.

## Methods

### Study design

Seabass fingerlings were purchased from MARESA (Spain) and transported to the Aquatic Engineering laboratory of ICBAS (Porto, Portugal). Fish were then acclimated to the experimental conditions for two-weeks while fed the control diet. Afterwards fish were individually weighed (initial body weight: 11.95 ± 0.34 g) and distributed in eight circular tanks of 80 L capacity with 30 fish per tank. Four tanks were fed with the control diet and four with the diet containing 5% supplementation with *Gracilaria* sp. For the first 80 days, tanks were connected to a closed recirculation seawater system ensuring similar quality parameters for all replicates. After this 80-day feeding period, all fish from 2 tanks from each diet (GRA or CTRL) were infected by injection with Phdp, whereas the fish from the 2 remaining tanks of each diet were administered a placebo injection. From inoculation time the tanks were individualized to prevent cross contamination. Water conditions were optimized and monitored daily to assure 30‰ salinity and 22 ± 0.5 °C temperature. A representation of the experimental design and the experimental units used in this study are summarized in Fig. S1.

### Experimental diets

Two isoproteic (50% DM) and isolipidic (19%) diets were distributed in four replicate tanks: a control diet (CTRL) and a supplemented diet with 5% *Gracilaria* sp. aqueous extract (GRA). The 5% supplementation level was selected based on previous works from the authors^26^ and relevant publications in the field^27,28^. *Gracilaria* sp. was produced by ALGAPlus in a land based Integrated Multitrophic Aquaculture (IMTA) system^29^. The seaweed was dried and thermally processed, using hot water at 83 °C for 160 min. After filtration, the resulting agar was recovered through a freeze-thawing process. The final solid product was washed, dehydrated with ethanol and dried at 60 °C overnight under vacuum. The extract was then added as supplement to the experimental diet at 5% w/w base, adjusted for dry matter (DM) content. All ingredients were finely ground (hammer mill, 0.8 mm sieve), mixed and then extruded (twin screw extruder, 2.0 mm pellet size, SPAROS, Portugal). Diets were finally dried at 45 °C for 12 h and stored at 4 °C until used. The detailed mineral and chemical compositions of the diets are presented in Table S1 of supplementary materials.

### Bacterial suspension and dose validation

*Photobacterium damselae* subsp. p*iscicida* (Phdp), strain SK-223/04, was purchased from CECT (Valencia, Spain). The strain was activated in tryptic soy broth (TSB; Biokar Diagnostics, France) and marine agar (Conda S.A., Spain). The bacteria were grown in TSB for 48 h at 22 °C until reaching the exponential phase. The inocula were then centrifuged at 3500 *g*, for 30 min at 22 °C and the pellet resuspended in NaCl 0.9% (Sigma-Aldrich). From this initial suspension, serial dilutions were performed to establish a Phdp growth curve and calculate inocula concentration. These dilutions were spectrophotometrically measured at 600 nm and plated in marine agar (incubation for 48 h at 22 °C) to count the number of colony forming units (CFU) and correlate the CFU counts with bacterial turbidity.

A dose validation trial was performed to establish an appropriate concentration for the infection. Surplus seabass were randomly distributed in tanks (10 fish per tank), anesthetized by immersion in 0.5 ml. l^−1^ of 2-phenoxietanol (Sigma-Aldrich) and intraperitoneally (i.p.) injected with 100 µl of saline solution (negative control) or Phdp suspension (3 test concentrations: 1.0 × 10^4^, 1.0 × 10^5^ and 1.0 × 10^6^ CFU ml^−1^). The survival rate was monitored for 7 days post-injection, which was the period that corresponded to the second consecutive day without mortalities. Samples were taken aseptically from the kidney of the infected fish, inoculated in marine agar plates and incubated 48 hours at 22 °C for CFU counts.

The method described by Reed and Muench^30^ was used to calculate endpoints, although for ethical reasons concerning animal welfare guidelines, the concentration of the inocula for the bacterial infection was calculated to achieve a lethal dose (LD) of 30 to 40% efficiency.

### Infection

After 80 days, 30 fish per tank from two replicate tanks fed on each of the diets were anesthetized and intraperitoneally injected with either a saline solution or Phdp suspension, similarly to the dose validation protocol described above. The Phdp inocula concentration was determined by absorbance (OD) as 5.93 × 10^6^ CFU.ml^−1^.

For the following 10 days tanks were closely monitored to account for mortality and feed intake. Afterwards all fish were weighted and 10 fish per tank were selected based on apparent good health and normal swimming and feeding behaviours. These fish were anesthetized by immersion in 0.5 ml. l^−1^ of 2-phenoxietanol (Sigma-Aldrich), blood was collected from the caudal vein and plasma obtained by centrifugation (5 min, 10000 g), aliquoted and stored at −80 °C. Liver, head kidney and spleen were also collected, and immediately frozen in liquid nitrogen and stored at −80 °C. Fish body weight and feed intake were calculated for the entire experimental period using the tank as experimental unit. The following formula was used to calculate Voluntary feed intake: 100 × [feed intake (g)/ABW (g)/trial duration (days), where ABW is ((IBW + FBW)/2). FBW and IBW are the initial and final average body weights (g).

### Plasma metabolites analysis

Plasma glucose (Glucose-RTU kit, Spinreact) and triglycerides (Triglycerides–LQ kit, Spinreact) concentrations were measured using 10 μL of plasma and the commercial kits adapted to microplate format, according to the recommendations of the manufacturer. Samples (8 fish per tank, N = 16) were evaluated in duplicate and blanks were performed for standardization.

### Immune plasma parameters

Immune parameters in plasma were assessed in 8 fish per tank (N = 16). Plasma lysozyme activity was evaluated by turbidimetric assay, according to Ellis^31^, based on the addition of the samples to a standard bacterial suspension of *Micrococcus lysodeikicus*. The absorbance decrease caused by bacterial lysis was measured by readings at 0.5 min and 4.5 min after addition. Values were standardized using hen egg white lysozyme (Sigma, Portugal). Plasma peroxidase levels were determined by the chemical reduction of 3,3_, 5,5_—tetramethyl benzidine hydrochloride (Sigma, Portugal), according to Quade and Roth^32^.

### Liver enzymatic assays

Livers were collected from 8 fish per tank (N = 16) and homogenised in phosphate buffer, 0.1 M pH 7.4. Part of the homogenates was used for analysis of thiobarbituric acid reactive substances (TBARS). The remaining portion was centrifuged at 10000 g for 20 min and supernatants used for analysis of protein content, catalase (CAT) and glutathione transferase (GST). Lipid peroxidation was measured by TBARS using methods described by Ohkawa, *et al*.^33^ and results were reported as nmol. g tissue^−1^. The protein content of homogenates was measured using methods described by Bradford^34^. CAT activity was determined according to methods described by Clairborne^35^ with hydrogen peroxide (30%) as substrate. GST activity was determined spectrophotometrically at 340 nm using 1-chloro-2,4-dinitrobenzene as substrate, according to the method described by Habig, *et al*.^36^. CAT and GST were reported as nmol. mg protein^−1^.

### Gene expression analyses

After homogenization with TRI reagent, total RNA from target tissues (liver, head kidney and spleen, N = 20) was extracted with MagMax-96 for microarrays total RNA isolation kit (Life Technologies, Carlsbad, USA). RNA quantity and purity were determined by Nanodrop (Thermo Scientific) with absorbance ratios at 260 nm/280 nm above 1.9. Reverse transcription (RT) of 500 ng of total RNA was performed with random decamers, using the High-Capacity cDNA Archive Kit (Applied Biosystems, USA). RT reactions were incubated for 10 min at 25 °C and 2 h at 37 °C. Real-time quantitative PCR (qPCR) was performed using an Eppendorf Mastercycler Ep Realplex Real-Time PCR Detection System (Eppendorf, Germany), using 96-well PCR array layouts designed for the simultaneous profiling of 19 genes in liver (Table S3) and 29 genes in head kidney and spleen (Table S4).

Genes selected for analysis in the liver were focused on oxidation-reduction processes, cell redox homeostasis, response to oxidative stress and cellular respiration. Genes selected for immune response evaluation in head kidney and spleen were involved in response to bacterium, cytokine-cytokine receptor interaction, cytokine signalling, response to cytokines and cell proliferation. Primers were designed to obtain amplicons of 50–150 bp in length. The arrays included 23 new sequences for seabass, already represented in the IATS-nutrigroup seabass transcriptomic database (www.nutrigroup-iats.org/seabassdb ^37^) and deposited in GenBank with the accession numbers MG596338-MG596342, MG596345, MH138003-MH138019 (Table S5). Among them, 16 are full codifying sequences. The PCR program used included an initial denaturation step (95 °C for 3 min), followed by 40 cycles of denaturation (15 s at 95 °C) and annealing/extension for 60 s at 60 °C. All pipetting operations were performed by an EpMotion 5070 Liquid Handling Robot (Eppendorf, Germany) to improve data reproducibility. PCR efficiency (between 90–100%) and reactions specificity were verified by melting curve analysis (ramping rates of 0.5 °C/10 s over a temperature range of 55–95 °C) and linearity of serial dilutions of RT reactions. Each sample was tested in triplicate and the fluorescence data acquired during the extension phase was normalized by the delta– delta Ct method^38^ using β-actin as the housekeeping gene.

### Statistical analysis

Statistical analyses were performed after all data was checked for normality (Shapiro-Wilk test) and homogeneity of variances (Levene’s test). The analysis of variance was performed applying two-way ANOVA test, with diet (CTRL and GRA) and infection (*Phdp*) as independent factors. Significant differences were considered for p < 0.05. The statistic software package used was SigmaPlot 12 (Systat Software Inc., U.S.A.) and information regarding experimental unit and N is presented in Supplementary Fig. 1 (Fig. S1). Multivariate analysis (Partial Least Squares-Discriminant Analysis; PLS-DA) was also performed to find discriminative features among groups by means of the EZ-Info software (Umetrics, Sweden). The contribution of genes in the PCR-arrays to the PLS-DA models was assessed by means of variable importance in projection (VIP) measurements. A VIP score > 1 was considered an adequate threshold to determine discriminant variables in the PLS-DA model^39,40^.

## Results

### Fish performance and mortality

Seabass showed no differences between dietary groups for initial and final body weight, as well as for feed intake calculated at day 90 (Supplementary Table S2). After infection fish were monitored daily and all mortalities recorded (Table 1). Placebo fish from both GRA and CTRL diets presented 100% survival rates. Regarding fish infected with Phdp, the first mortalities occurred in the CTRL diet, specifically at day 1 and day 3 post-infection. At day 3, mortalities were observed in both dietary groups infected with Phdp. Overall, deaths occurred within the predicted time-line for bacterial infections, i.e. between days 3 and 7 post inoculation.

**Table 1:** Mortality and survival percentage recorded for 10 days post-infection in seabass fed the experimental diets (CTRL or GRA) and subjected to *Phdp* infection.

Days Post-Infection												
DIET_INFECTION	1	2	3	4	5	6	7	8	9	10	Total Dead	% survival
CTRL_PLACEBO	0	0	0	0	0	0	0	0	0	0	0	100.00^a^
CTRL_PLACEBO	0	0	0	0	0	0	0	0	0	0	0	100.00^a^
GRA_PLACEBO	0	0	0	0	0	0	0	0	0	0	0	100.00^a^
GRA_PLACEBO	0	0	0	0	0	0	0	0	0	0	0	100.00^a^
CTRL_PHDP	0	0	2	0	1	2	1	0	0	0	6	80.00^b^
CTRL_PHDP	1	0	1	2	1	0	2	0	0	0	7	76.67^b^
GRA_PHDP	0	0	0	2	0	0	4	0	0	0	6	80.00^b^
GRA_PHDP	0	0	2	0	0	0	0	0	0	0	2	93.10^b^

N = 2 tanks per group. Superscript letters indicate significant differences between infected and placebo groups (p < 0.05).

### Plasma bioindicators

**Figure 1:**
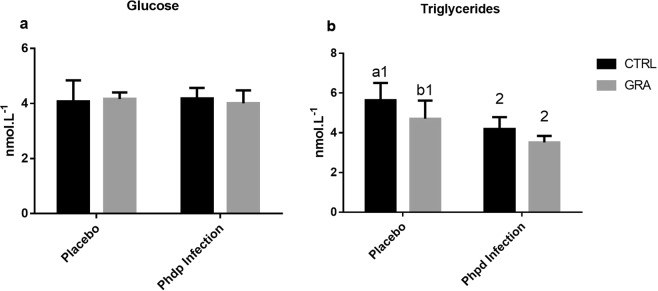
Glucose (**a**) and Triglycerides (**b**) levels analysed in plasma of seabass fed the experimental diets (CTRL or GRA) and subjected to *Phdp* infection. Results presented as mean ± standard deviation. N = 16 fish per group. Different letters indicate significant differences between diets and different numbers indicate differences between infection and placebo (p < 0.05).

The evaluation of glucose levels (Fig. 1a) revealed no differences between diets, infection or the interaction between both factors. On the contrary, triglycerides levels (Fig. 1b) within placebo groups (p = 0.018), showed lower triglycerides levels in seabass fed GRA compared to CTRL and no differences were detected between diets within Phdp infected fish. In addition, within each diet, highly significant decreases were detected in Phdp infected fish when compared with placebo (p < 0.001). No statistical significance was found for the interaction between diet and infection

### Immune parameters in plasma

**Figure 2:**
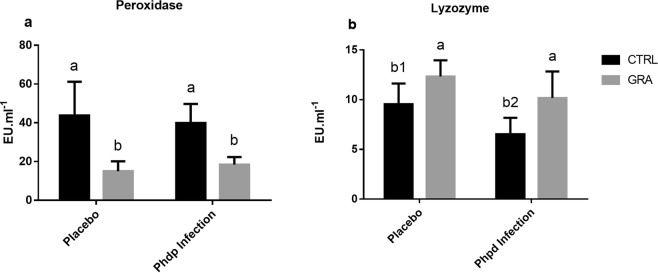
Peroxidase (**a**) and lysozyme (**b**) activities determined in plasma of seabass fed the experimental diets (CTRL or GRA) and subjected to *Phdp* infection. Results presented as mean ± standard deviation. N = 16 fish per group. Different letters indicate significant differences between diets and different numbers indicate differences between infection and placebo (p < 0.05).

The measurement of circulating peroxidase activity levels (Fig. 2a) revealed differences between dietary treatments (p < 0.05) and an interaction between diet and infection (p < 0.05). Specifically, fish fed GRA diet presented lower peroxidase levels in both placebo and Phdp groups when compared with these fed the CTRL diet. Lysozyme activity (Fig. 2b) showed statistical differences between dietary groups with higher levels observed for GRA diet (p < 0.001) and lower levels in Phdp groups when compared with placebo (p = 0.004). Additionally, the interaction of both factors (diet and infection) evidenced that seabass fed the CTRL diet had lower lysozyme activity (p = 0.005) than fish fed GRA diet.

### Antioxidant parameters in liver

**Figure 3:**
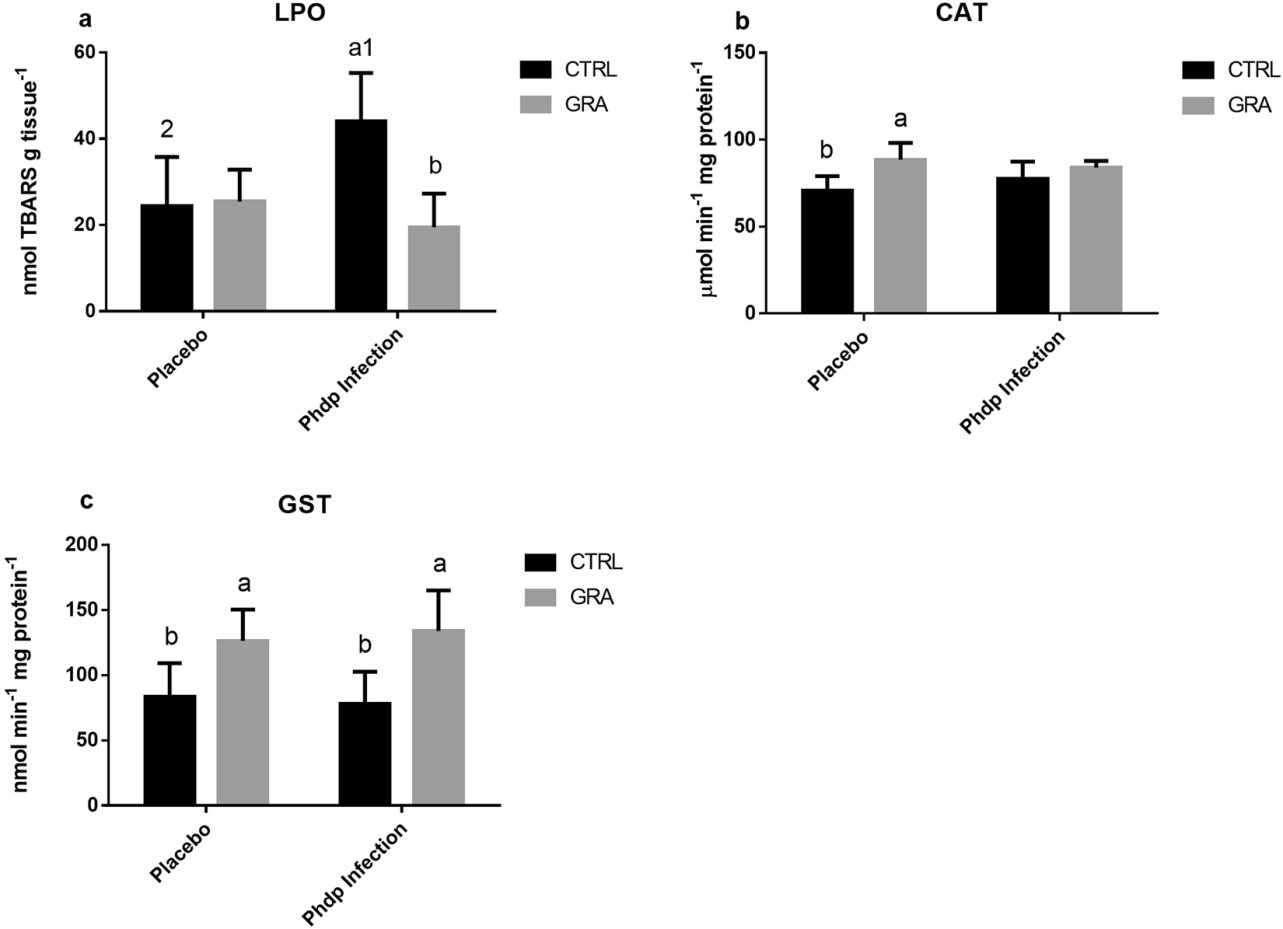
Lipid peroxidation (**a**), Catalase (**b**) and glutathione s-transferase (**c**) activities determined in liver of seabass fed the experimental diets (CTRL or GRA) and subjected to *Phdp* infection. Results presented as mean ± standard deviation. N = 16 fish per group. Different letters indicate significant differences between diets and different numbers indicate differences between infection and placebo (p < 0.05).

Lipid peroxidation (Fig. 3a) displayed significant differences between diets within the Phdp infected groups, with lower peroxidation in seabass fed GRA diet (p = 0.018). Considering the CTRL diet alone, seabass infected with Phdp revealed higher lipid peroxidation (p = 0.002). Catalase activity (Fig. 3b) was higher in fish fed GRA diet within the placebo groups (p = 0.002), and no statistical differences were detected between diets in Phdp infected groups or the interaction of both diet and infection. Glutathione s-transferase activity (Fig. 3c) significantly increased (p < 0.001) in fish fed GRA diet in both placebo and Phdp groups.

### Gene expression analysis

The effects of diet and infection were assessed using specific PCR-arrays focused on genes related to cell redox homeostasis and response to oxidative stress in liver, or immune response and cell proliferation in head kidney and spleen. The expression results for each experimental group and tissue, and the corresponding two-way ANOVA analysis can be found in Supplemental Tables S6–S8. The effect of diet was more evident in liver and head kidney comparatively to the effect of the Phdp infection. In the liver, the expression of 13 out of 19 genes was found to be differentially modulated by diet, whereas only 6 genes were differentially expressed due to infection, and 7 genes by the combination of both factors. In head kidney, diet led to the differential expression of 9 out of 29 genes, while 4 genes were affected by the infection, and the combination of diet and infection contributed to the differential expression of 7 genes. The relative effect of diet was less evident in the spleen, as the same number of genes, 7, was differentially expressed by diet or infection, whereas their combination affected 3 genes.

**Figure 4:**
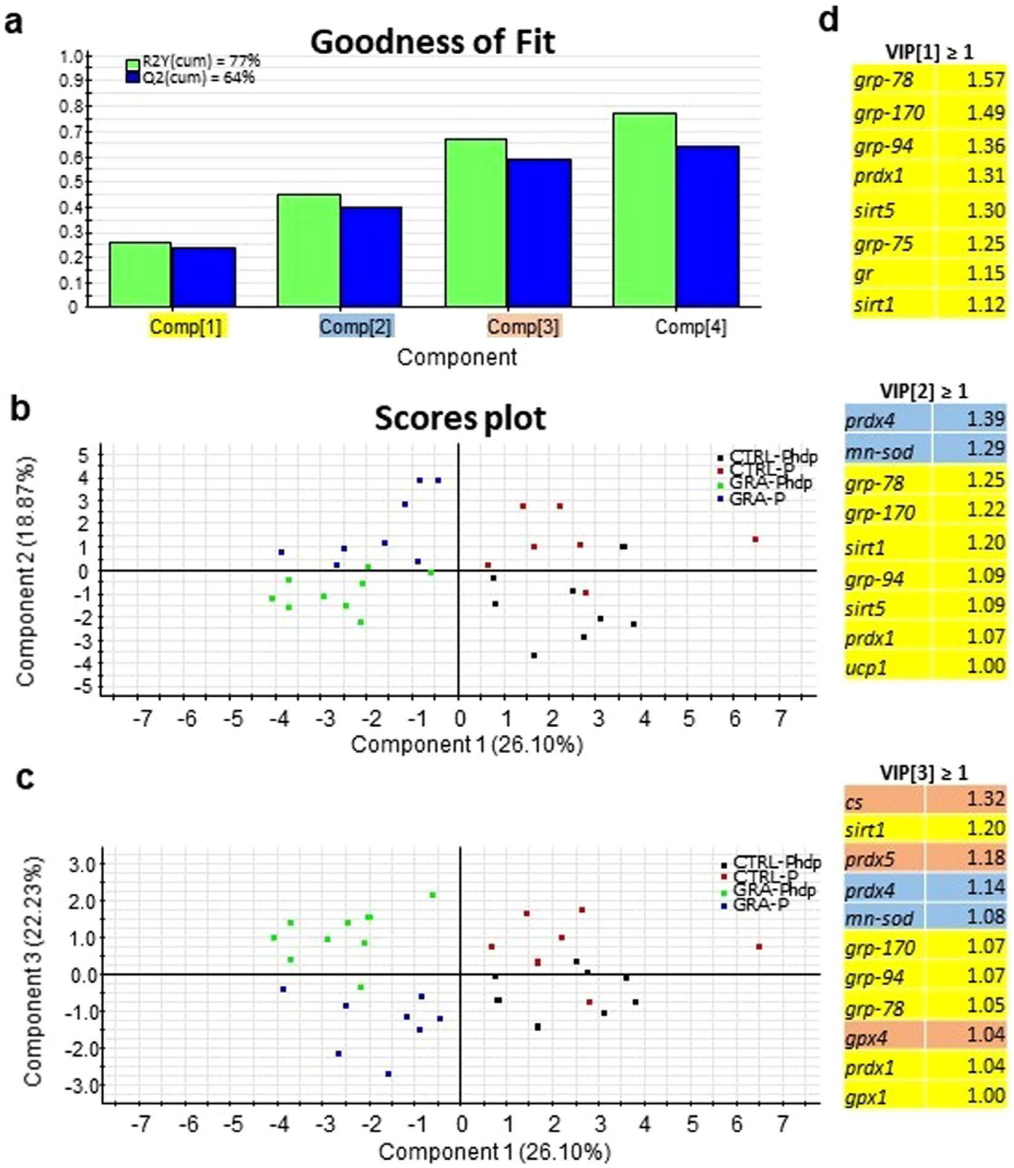
Discriminant analysis (PLS-DA) of liver molecular signatures of seabass altered by dietary *Gracilaria* sp. supplementation and/or Phdp infection (N = 20 fish per group). (**a**) Cumulative coefficients of goodness of fit (R2, white bars) and prediction (Q2, grey bars) by each component; 77% of total variance is explained by four components. (**b)** PLS-DA score plot of acquired data from infected individuals along component 1 and 2. (**c**) PLS-DA score plot of acquired data from infected individuals along component 1 and 3. (**d**) Ordered list of markers by variable importance (VIP) in the projection of PLS-DA model for group differentiation. Markers with VIP values > 1 after the first, second and third components are highlighted in yellow, blue and orange, respectively.

A multivariate analysis approach was used to visualize the interplay between diet and infection. For instance, in liver, 77% of total variance was explained by 4 components, with the 3 main components defining more than 67% of variance (Fig. 4a). Seabass fed CTRL and GRA diets were clearly separated within the first component (26.10% of total variance), whereas separation along component 2 (18.87% of total variance, Fig. 4b) and component 3 (22.23% of total variance, Fig. 4c) contributed to the differentiation of infected and non-infected fish within each dietary group. This approach also revealed which genes presented a higher contribution to variation (VIP ≥ 1, Fig. 4d). The main contributors to component 1, which reflected the effect of diet were genes encoding heat shock proteins (*grp-78*, *grp-170*, *grp-94*, *grp-75*) that were found to be down-regulation in seabass fed GRA diet, together with *prdx1*, *sirt5*, *gr* and *sirt1*. VIP contribution of 2 components highlighted that the differential response to Phdp infection was mainly due to the down-regulation of *prdx4* and *mn-sod*. Separation along component 3 revealed the contribution of *cs*, *prdx5* and *gpx4*, and for the 2 later genes no significant effect of diet or infection was found, but a significant interaction of both factors was evidenced.

**Figure 5:**
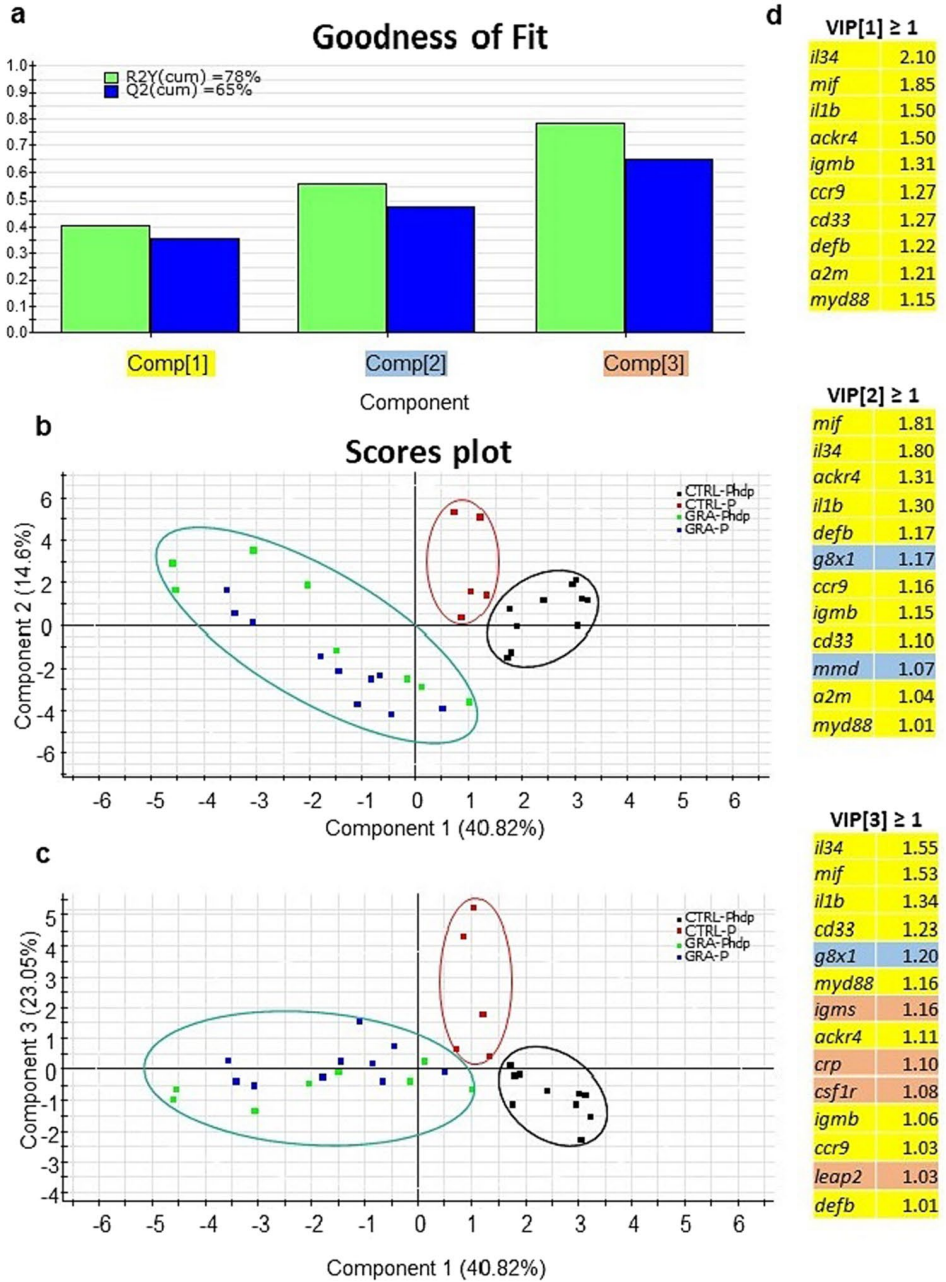
Discriminant analysis (PLS-DA) of head kidney molecular signatures of seabass altered by dietary *Gracilaria* sp. supplementation and/or Phdp infection (N = 20 fish per group). (**a**) Cumulative coefficients of goodness of fit (R2, white bars) and prediction (Q2, grey bars) by each component; 78% of total variance is explained by four components. (**b**) PLS-DA score plot of acquired data from infected individuals along component 1 and 2. (**c**) PLS-DA score plot of acquired data from infected individuals along component 1 and 3. (**d**) Ordered list of markers by variable importance (VIP) in the projection of PLS-DA model for group differentiation. Markers with VIP values > 1 after the first, second and third components are highlighted in yellow, blue and orange, respectively.

PLS-DA analysis of transcriptional response along component 1 in head kidney also highlighted a clear separation regarding diets (40.82% of total variance) (Fig. 5b). Scores of components 1 and 2 allowed to discriminate between infected and non-infected individuals fed the CTRL diet (Fig. 5c), although this separation was better accomplished along component 3, which explains 23.05% of total variance (Fig. 5d). In seabass fed the GRA diet, infected and non-infected individuals overlapped in all scores and were therefore analysed together. With this approach, the three components explained 78% of total variance (Fig. 5a). The most relevant VIP in component 1 for fish fed GRA diet revealed the contribution of several genes in group separation via up-regulation (*il34*, *ccr9*, *cd33*) or down-regulation (*mif*, *il1b*, *defb*, *a2m*, *myd88*). VIP analysis after two components highlighted the role of *g8x1* and *mmd*, which were down-regulated with infection in individuals fed CTRL diet. This separation was more evident with component 3, in which a clear up-regulation of *IgMs* was evident in infected fish of both dietary groups. The down-regulation of *leap2* caused by Phdp infection was also highlighted as a relevant contributor for VIP after 3 components, regardless of the low expression level of this gene in head kidney.

Regarding spleen, PLS-DA analysis only discriminated two components, with low supported (30%) or predicted (11%) variance and with no clear separation among experimental groups in the scores plot (Supplemental Fig. S2).

## Discussion

Dietary supplementation has emerged as an indispensable tool to improve fish health either by boosting immunity, using specific molecules such as β-glucans, or by providing readily available antioxidants such as vitamins. In the present work we evaluated the contribution of dietary 5% *Gracilaria* sp. aqueous extract supplementation in seabass immune and antioxidant capacities when infected with Phdp.

In the present work seabass weight and feed intake showed no differences between diets supporting the use of *Gracilaria* sp. at 5% supplementation level. These results were calculated at the end of the experiment since weighing procedures would act as an abiotic stressor compromising the results of the bacterial infection^41^. Nevertheless, the absence of differences in FBW and VFI between fish fed the CTRL and GRA diets is in accordance with previous tests conducted in seabass using the inclusion of two different *Gracilaria* species (*G*. *bursa-pastoris* and *G*. *cornea*) at 5 and 10% levels, in which no negative consequences on growth performance, nutrient utilisation and body composition were detected^22^. Furthermore, within placebo groups, GRA diet significantly lowered triglycerides levels, suggesting that *Gracilaria* sp. supplementation modulates seabass fatty acid metabolism pathways. However, Phdp overrode the effect of diet, consequently no differences were observed in glucose and triglycerides levels after infection. The results obtained in placebo groups substantiate the antihyperlipidemic effect described in rats^42^, mice^43^, chickens^44^ and zebrafish^43^ when fed diets supplemented with seaweeds, as well as the lower cholesterol and triglyceride levels observed in Japanese flounder (*Paralichthys olivaceus*) fed diets supplemented with *Eucheuma denticulatum*^45^ and barramundi (*Lates calcarifer*) fed diets containing *Gracilaria pulvinate*^46^.

Regarding mortality, our results validate the use of *Gracilaria* sp. supplementation as an effective tool to delay photobacteriosis since groups fed the CTRL diet registered the earliest deaths. Additionally, despite no significant differences in cumulative mortality, the total number of dead fish was lower in GRA group. Similar results have been found by Van Doan, *et al*.^47^ when feeding basa fish (*Pangasius bocourti*) with diets supplemented with agar. Also, improved resistance to vibriosis was described by Castro, *et al*.^48^ when treating turbot (*Scophthalmus maximus*) phagocytes with seaweed water-soluble extracts.

The current work also intended to evaluate the mechanisms through which *Gracilaria* sp. supplementation nutritionally-modulated fish resistance to infection, by accessing the innate immune indicators lysozyme and peroxidase. Both indicators were selected since macrophages engage immediately after infection in defence mechanisms releasing the peroxidases stored in their cytoplasmic granules^49^, causing an initial increase in plasmatic levels that progressively decreases over time^50^. The current study showed that peroxidase levels decreased in both infected and placebo groups fed GRA diet, leading to the hypothesis that GRA may elicit an immediate response, which was no longer detectable 10 days post infection. Leukocytes also respond to bacterial infection by releasing lysozyme, an enzyme with lytic activity against pathogens^49^ and is overexpressed in the presence of microbial agents or after immunization procedures^51^. In the current study, lysozyme activity increased in seabass fed GRA diet compared with fish fed CTRL, suggesting a boosting effect possibly triggered by polysaccharides present in the seaweed-supplemented diet. Considering the current results, it is plausible to infer that dietary *Gracilaria* sp. modulates seabass immune system, eliciting a primary response, which may be advantageous to delay photobacteriosis.

In infectious milieus, activated phagocytes increase ROS production whose microbicidal properties are important to degrade pathogens^48^. However excessive ROS production can lead to oxidative stress, a phenomena previously described in infection scenarios^52^. In the current study, dietary *Gracilaria* sp. may play a role maintaining the redox balance after infection, as lower lipid peroxidation values were detected in infected fish fed GRA when compared to these fish fed the CTRL diet. Our results are in line with the decrease in lipid peroxidation products observed in rainbow trout (*Oncorhynchus mykiss*) when fed diets supplemented with *Gracilaria pygmaea*^53^. Further indication of an upregulated detoxifying activity in seabass fed GRA diet was provided by glutathione S-transferase (GST) analysis, as GST activity was increased in both infected and placebo groups fed that diet. Recently Thanigaivel, *et al*.^54^ have shown that the antioxidant response of *Oreochromis mossambicus* to a bacterial infection with Aeromonas is improved in fish fed microencapsulated extracts of *Gracilaria foliifera* and *Sargassum longifolium*.

The reported changes in plasma bioindicators and enzyme activities in response to GRA supplementation and/or bacterial infection pointed towards differential expression signatures in fish facing these challenges. Indeed, in the liver of seabass fed GRA diet, a clear decrease was detected in the expression of genes encoding heat shock proteins and molecular chaperones *i*.*e*. *grp94*, *grp170*, *grp78*, *grp75* and *mthsp10*, established markers of fish response to stressors^55^. Besides their involvement in stress responses, heat shock proteins are also involved in immunity processes^56,57^ playing a major role mediating the development of inflammation through specific and non‐specific responses to infections^58^. *Gracilaria* sp. supplementation also affected the hepatic expression of antioxidant enzymes, either inducing a down-regulation (glutathione reductase, *gr*; peroxiredoxin 1, *prdx1*) or a reverse response to bacterial infection (glutathione peroxidase 4, *gpx4*; peroxiredoxin 5, *prdx5*) in comparison to CTRL. Considering their function in cell defence mechanisms as modulators of inflammation and cell protection^59,60^, the down-regulated expression suggests a protective role of *Gracilaria* sp. in seabass response to pathogens. Moreover, fish sirtuins (*sirt*) were already demonstrated to respond to dietary changes^61^ and in our results *sirt 1* and 5 hepatic expression levels showed an inverse pattern between dietary groups infected with Phdp. These results seem to follow the same mechanistic regulation as in mammals, where *sirt1* inhibition is associated to *sirt5* overexpression and are linked with the resolution of inflammation^62^. Globally, genes encoding for antioxidant enzymes and redox homeostasis were down-regulated in the liver of seabass fed GRA diet, suggesting a direct contribution of *Gracilaria* sp. to the antioxidant processes, dismantling the need for increased transcription.

Head-kidney gene expression analysis also revealed that GRA diet modulated the expression of cytokines, key regulators of infection^63^, especially when infected with Phdp. Palstra, *et al*.^64^ also found improved chemotaxis and chemokine-mediated signalling in the defence against Gram-positive bacterium in *Salmo salar* fed diets supplemented with *Laminaria digitata*. In our work, seabass fed GRA diet showed a decreased expression of the pro-inflammatory cytokine *il-1β* at 10 dpi, and since this cytokine acts as a chemoattractant for leucocytes, which involves the recruitment of other interleukins^65^, its decreased transcription may represent the resolution of inflammation. In line with this assessment, IL-1β was reported to enhance macrophage functions in seabass infected with *Vibrium anguillarum* immediately after infection^66^ and to decrease expression in rainbow trout (*Oncorhynchus mykiss*) head kidney 8 days after exposure to *Aeromonas salmonicida*^67^. Likewise, in our work the anti-inflammatory cytokines *il-10* and *il-20* increased in seabass fed GRA and infected with Phdp. IL-20 in fish has been identified as belonging to the IL-10 family, and both these cytokines were previously observed to increase in fish macrophages after infection with *Yersinia ruckeri*^68^. Additionally, the transcription levels of *il-34* together with *csf1r*, and *cd33* increased in infected seabass fed GRA diet, suggesting higher involvement of macrophages in defence against Phdp.

The influence of dietary *Gracilaria* sp. supplementation in seabass is further evidenced by the up-regulation of the lymphokine *mif*, the chemokine receptor *ackr4* and the antimicrobial peptide *defb* in response to infection, all involved in immunosuppression^65^. Immunoglobulins (Ig) which are involved in both innate and adaptive immunity showed increased expression in infected fish fed GRA diet. IgM has been described to activate complement and lysozyme triggering the lysis and opsonisation of pathogens^69,70^, and also mediate agglutination, phagocytosis and pathogen removal^71^. Therefore, the observed up-regulation of *igm* together with the increased lysozyme activity, further supports the hypothesis that dietary *Gracilaria* sp. supplementation positively regulates seabass resistance to Phdp. Overall, the up-regulated expression in immune related genes observed in seabass fed GRA diet when compared to CTRL suggests heightened immunity against infection with Phdp.

To summarize, our results show that a dietary supplementation of 5% aqueous extract of *Gracilaria* sp. is feasible for seabass without compromising weight gain or affecting feed consumption rates. Moreover, when infected with *P*. *damselae*, seabass fed GRA diet were more resistant to the pathogen, constituting an advantageous feature in aquaculture industry. Additionally, lysozyme and peroxidase in seabass fed GRA revealed increased resistance to pathogen proliferation. The protective role of *Gracilaria* sp. in oxidation processes resulted in lower LPO levels and increased GST activity, suggesting amplified capacity to respond to higher ROS levels produced during inflammation. Seabass fed GRA diet also evidenced differential expression of key genes involved in the immune and antioxidant systems when compared to the CTRL. More specifically, a shift in the contribution of determinant genes for the inflammatory process was observed in GRA groups evidencing a determinant role of *Gracilaria* sp. supplementation in the up-regulation of immune and antioxidant related pathways.

### Ethical statement

All procedures were conducted under the supervision of an accredited expert in laboratory animal science by the Portuguese Veterinary Authority (1005/92, DGV-Portugal, following FELASA category C recommendations), according to the guidelines on the protection of animals used for scientific purposes from the European directive 2010/63/UE. The experiment took place at the Abel Salazar Biomedical Sciences Institute (ICBAS), University of Porto (Portugal). This study was approved by the ORBEA (Organismo Responsável pelo Bem-Estar dos Animais), the Institutional Animal Care and Use Committee (IACUC) of ICBAS.

## Supplementary information

Supplementary data

## Data Availability

The datasets generated during the current study are available on request to the corresponding author.
