# Supplementary material for: Protective effects of seaweed supplemented diet on antioxidant and immune responses in European seabass (Dicentrarchus labrax) subjected to bacterial infection
Source: Sci Rep. 2019 Nov 6;9:16134. doi: 10.1038/s41598-019-52693-6 (PMC6834676; doi:10.1038/s41598-019-52693-6)
Supplement: Supplementary file 1 — Supplementary data [file 41598_2019_52693_MOESM1_ESM.pdf]

Protective effects of seaweed supplemented diet on antioxidant and immune responses in European seabass (*Dicentrarchus labrax*) subjected to bacterial infection

Maria J. Peixoto[a,b], Renato Ferraz[a,b], Leonardo J. Magnoni[a,e], Rui Pereira[c], José F. Gonçalves[b], Josep Calduch-Giner[d], Jaume Pérez-Sánchez[d], Rodrigo O. A. Ozório[a,b]*

[a] - CIIMAR – Centro Interdisciplinar de Investigação Marinha e Ambiental, Universidade do Porto, Rua dos Bragas 289, 4050-123 Porto, Portugal.

[b] - ICBAS – Instituto de Ciências Biomédicas de Abel Salazar, Universidade do Porto, Rua de Jorge Viterbo Ferreira 228, 4050-313 Porto, Portugal.

[c] - ALGAPLUS, Lda - Travessa Alexandre da Conceição S/N., 3830-196 Ílhavo, Portugal.

[d] - Nutrigenomics and Fish Growth Endocrinology Group, Institute of Aquaculture Torre de la Sal, IATS-CSIC, 12595 Ribera de Cabanes, Castellón, Spain.

[e] - IIB-INTECH - Instituto de Investigaciones Biotecnológicas - Instituto Tecnológico de Chascomús (CONICET), Chascomús, Argentina.

*Corresponding author: Rodrigo O. A. Ozorio.
E-mail address: rodrigo.ozorio@ciimar.up.pt
CIIMAR - Centro Interdisciplinar de Investigação Marinha e Ambiental, Universidade do Porto, Terminal de Cruzeiros do Porto de Leixões, Avenida General Norton de Matos, 4450-208, Matosinhos, Portugal.
Tel.: + 351 223401820

Supplementary Materials

Table S1 Proximate and chemical composition of the experimental diets.

**Ingredients (%)**	**CTRL**	**GRA**
Fishmeal 60	22.00	20.94
CPSP 90	3.00	2.85
Feather meal hydrolysate	5.00	4.75
Haemoglobin powder	5.00	4.75
Poultry meal 65	20.00	19.04
Pea protein concentrate	3.50	3.32
Soybean meal 48	9.00	8.56
Rapeseed meal	3.50	3.32
Wheat meal	14.80	14.09
Fish oil – SAVINOR	12.00	11.42
Vit & Min Premix PV01	1.00	0.94
Lutavit E50	0.03	0.02
Choline chloride	0.07	0.06
Betaine HCl	0.10	0.09
Binder (Kieselghur)	0.30	0.28
Antioxidant	0.20	0.18
Sodium propionate	0.10	0.09
L-Lysine	0.20	0.18
DL-Methionine	0.10	0.09
L-Taurine	0.10	0.09
Gracilaria sp.	0.00	5.00
**Chemical composition**	**CTRL**	**GRA**
Dry matter (%)	93.9 ± 0.0	93.5 ± 0.2
Crude protein (%DM)	51.4 ± 0.0	50.3 ± 0.1
Crude fat (%DM)	19.1 ± 0.3	18.2 ± 0.2
Gross Energy (kJ/g DM)	23.6 ± 0.1	23.4 ± 0.1
Ash (%DM)	10.2 ± 0.2	10.1 ± 0.1

* *Gracilaria* sp. extract was added as separate supplement to the basal diet at 5 % w/w base, adjusted for dry matter content (DM).

Table S2: Zootechnical parameters of seabass fed the experimental diets (CTRL or GRA) subjected infection with *Phdp*. Values presented as mean ± standard deviation.

	CTRL				*GRA*				*p - value*		
	Placebo		*Phdp*		*Placebo*		*Phdp*				
	AVG	*STD*	*AVG*	*STD*	*AVG*	*STD*	*AVG*	*STD*	*Diet*	*Infection*	*D x I*
IBW	11.92	0.63	12.20	1.16	11.64	0.26	12.05	0.43	0.44	0.22	0.83
FBW	28.00	1.57	26.83	4.55	28.58	0.19	28.43	2.49	0.23	0.35	0.44
VFI	1.77	0.07	1.87	0.06	1.80	0.08	1.81	0.08	0.92	0.52	0.38

N=2 tanks; 30 fish per tank. No significant differences were found for diet, infection or their interaction in two-way ANOVA for $p<0.05$.

IBW, Initial body weight (g); FBW, Final body weight (g); VFI, Voluntary feed intake (% body weight per day).

Table S3. Forward and reverse primers for the oxidative stress pathway focused PCR-array.

Gene name	Symbol		Primer sequence (5'-3')
β-actin	*actb*	F	TCC TGC GGA ATC CAC GAG A
		R	AAC GTC GCA CTT CAT GAT GCT
Citrate synthase	*cs*	F	GTG TAT GAG ACC TCC GTG TTG G
		R	AGC AAC TTC TGA CAC TCT GGA ATG
Cytochrome c oxidase subunit I	*coxi*	F	ATA CTT CAC ATC CGC AAC CAT AA
		R	AAG CCT CCG ACT GTA AAT AAG AAA
Mitochondrial respiratory uncoupling protein 1	*ucp1*	F	CGA TTC CAA GCC CAG ACG AAC CT
		R	TGC CAG TGT AGC GAC GAG CC
Sirtuin 1	*sirt1*	F	GGT GGA CCT CTT GAT TGT CAT TGG CTC TTC
		R	GGG ATG AGG GCA ACT GGT CGG ACT TTA
Sirtuin 5	*sirt5*	F	AGA CAC AGA TGA CGC AGA GAT
		R	TCA GGA GAC CGT GAC AGC
Catalase	*cat*	F	GCA TCA GGT GTC TTT CTT GTT CAG
		R	GGA GCC GTA GCC GTT CAT
Superoxide dismutase Mn	*mn-sod*	F	AGG CTA TCT GGA ATG TCA TCA ACT GGG AGA
		R	GCA GTC TGG AGA CGC TCG GTC AC
Glutathione peroxidase 1	*gpx1*	F	TGC CCA CCC TTT GTT TGT CTA TCT
		R	CCA TCA GGA CTG ACC AGG AAC T
Glutathione peroxidase 4	*gpx4*	F	GCA CGC CAA GTA CGC TGA GAG
		R	GCT CCT GGT TCC CAA ACT GGT TAG A
Glutathione reductase	*gr*	F	GGT GGA CTG TCT CCT GTG
		R	TCA TCT CGC CGA TGT TCA
Peroxiredoxin 1	*prdx1*	F	GTC TGG GTT CTA TGA AGA TTC CGC TGG TAT
		R	TTA GGA CGC CGT AAT CAG TGG AGA TG
Peroxiredoxin 4	*prdx4*	F	TGT TGA CTC CCA GTT CAC CCA CCT T
		R	CCA CCC TGC TTC CTC GGC GTA T
Peroxiredoxin 5	*prdx5*	F	TGT TGG TGG AAG ATG GAG TTG TGA AGA AGA
		R	CAG GTC AGC CCA GTG CCA TCA G
Peroxiredoxin 6	*prdx6*	F	CCA CCC AAG GGA CTT CAC
		R	CTG ATC CTA GCG GCA CAG
Glucose-regulated protein 94	*grp-94*	F	CGA TGG CAC AGT AGA AGA GGA CCT TGG TAA
		R	GCA CCG CCT CAT CAT CTG TTC TGG
Glucose-regulated protein 170	*grp-170*	F	CTA CGG CGA CCT CAG CTT CCT
		R	GTC AGG TTC AGA GAG CCA AAC ACA CT
Glucose-regulated protein 78	*grp-78*	F	GCC GAC GAC GAC GAT AAG AGG
		R	CCAG GTC GAT TCC AAC CAC AGT
Mitochondrial Hsp10	*mthsp10*	F	GCT TTA CTC CAG AGA CTG TGA CGA A
		R	CAG CAC CTT GCC TTG AGA CTT C
Mitochondrial Hsp70 mortalin	*grp-75*	F	GCT GCT GGT CGG AGG AAT GTC
		R	GTC CTG AAC CGT TTG CTG AAC CTT

Table S4. Forward and reverse primers for the immune pathway focused PCR-array.

Gene name	Symbol		Primer sequence (5'-3')
β-actin	*actb*	F	TCC TGC GGA ATC CAC GAG A
		R	AAC GTC GCA CTT CAT GAT GCT
Interleukin 1-beta	*il1b*	F	CAT GAG CGA GAT GTG GAG ATC CAA GAT
		R	CAT TGT CAG TGG GTG GTG GGT AAT C
Interleukin-8	*il8*	F	CAA TCA GCA GGG ACT ACA ACA CAC A
		R	CTG TCT GGA GGG ATG ATC CTT GAC T
Interleukin-10	*il10*	F	CAG TGC TGT CGT TTT GTG GAG GGT TTC
		R	TCT CTG TGA AGT CTG CTC TGA GTT GCC TTA
Interleukin-20	*il20*	F	GCT AGA AAT AAA GGA GGC GGC ACA GAA GG
		R	CAG TCC AGC ACA GTG TCC AGT TCT C
Interleukin-34	*il34*	F	AGA ACC CGA CAG AGT GCC AGA GT
		R	CAG GAG GGA TTT TGG GGA CGC ATA TC
Tumour necrosis factor α	*tnfα*	F	TCT ACA GCC AGG CGT CGT TCA G
		R	CCG CAC TTT CCT CTT CAC CAT CGT
C-C chemokine receptor type 3	*ccr3*	F	TGA CCT TCG ACC GAC ACC TA
		R	ACA ATA CAG GAG ACT ACC GCA TAG C
C-C chemokine receptor type 9	*ccr9*	F	CCT GTG TGT CTG GCT TGT TTC TAC TCT C
		R	TCG CTC TTC ACC TGG GCA AAG ATA AAC TC
Atypical chemokine receptor 4/C-C chemokine receptor type 11	*ackr4*	F	TAC TTC TCT TCA CCC TGC CTT TCT G
		R	GCT GCC GAA CCC AAC TTC CA
T-cell surface glycoprotein CD3 zeta chain	*cd247*	F	CTG ATG CGT CTG AAG AGA ATG GAG GC
		R	GTT CAA GCA CCT GGT AAG GAT CAG CAT C
T-cell surface glycoprotein CD8 beta	*cd8b*	F	AGT GAT CCC GCC AAC ATT ACC TCC TA
		R	TCT TCT TAG GGC AGC GAC AGA CT
Myeloid differentiation primary response protein MyD88	*myd88*	F	CCA ATT CAG GTT GAT GAG GTT GAC A
		R	TCC TCC AGG GTG ATA CCA ATC C
Myeloid cell surface antigen CD33	*cd33*	F	CTG TTC ATT CAC CCA TCC TAG AG
		R	GGT CGA ACG ATG CCA GAT T
Macrophage colony-stimulating factor 1 receptor	*csf1r*	F	CGG GCA GGA ACA GCT AAT CTA CCA
		R	ACT TGG GCT CAT CAC ACA CTT CAC
Macrophage migration inhibitory factor	*mif*	F	GCT CCC TCC ACA GTA TTG GCA AGA T
		R	TTG AGC AGT CCA CAC AGG AGT TTA GAG T
Monocyte to macrophage differentiation factor	*mmd*	F	GGT CAT CTA CTT CTT CAT CGC TGC CTC CTA
		R	CCA ACT CTC GCA GGT TCA ACC AAG GT
Interferon regulatory factor 8	*irf8*	F	TCT GAA GGC TGC CGA ATC TCC
		R	CTG TCT GAA CTG TAT AGG GCA CCA C
Nuclear factor NF-kappa-B p100 subunit	*nfkb2*	F	CTG GAG GAA ACT GGC GGA GAA GC
		R	CAG GTA CAG GTG AGT CAG CGT CAT C
Liver-expressed antimicrobial peptide 2	*leap2*	F	GGT TTG CTC CAA CGG ACC AA
		R	CAC AGG CTT CAT GCT GTT CCA
Lysozyme C	*lyz*	F	CGG AGC CAT CAA CCA CAA CAC TG
		R	GCC ATT ATT ACA CCA CCA GCG ACT GT
Beta-defensin	*defb*	F	GGG CTG AGC TTG GTT CTC CTT GT
		R	CCT CCC CAA CTG CGA GCA TCA
IgM membrane-bound	*igmb*	F	ACA GAG GAA GAT AAC ATG GCG GTG G
		R	TGG TTA CAA TGG TGA ACA GCA GAG TGAT

Table S5. Classification of new assembled sequences according to BLAST-X searches.

Contig	Size (nt)	Annotation[a]	Best match[b]	E[c]	CDS[d]	GenBank accession
L12_73806	1173	*leap2*	XP_023257275	1e-38	200-439	MG596338
L12_66506	950	*lyz*	KKF29953	7e-88	195-626	MG596339
L2_17762	328	*defb*	ADJ21805	7e-29	<1-226	MG596340
L12_87068	2152	*igmb*	ARC77253	0	139-1632	MG596341
L3_71324	569	*a2m*	XP_019123863	8e-90	<1->569	MG596342
L12_74857	1236	*lgals8x1*	ANN46245	0	43-999	MG596345
L12_80996	1495	*ucp1*	XP_023147774	0	162-1082	MH138003
L2_44162	707	*sirt1*	XP_018536491	1e-161	<1->681	MH138004
L12_83923	1727	*sirt5*	XP_008276398	0	65-982	MH138005
L12_87749	2310	*cat*	XP_022611780	0	<1-1047	MH138006
L3_68914	981	*mn-sod*	ANS56706	7e-164	51-728	MH138007
L12_69789	994	*gpx1*	XP_023265962	8e-124	227-655	MH138008
L3_67061	957	*gpx4*	XP_023118319	3e-122	54-629	MH138009
L12_86603	2069	*gr*	XP_018549649	0	83-1576	MH138010
L3_72270	1072	*prdx1*	XP_018532829	5e-136	61-657	MH138011
L12_66928	955	*prdx4*	XP_019941327	3e-172	92-883	MH138012
L12_67332	960	*prdx5*	XP_018547633	3e-129	131-703	MH138013
L3_49822	768	*prdx6*	XP_018525676	3e-155	79-744	MH138014
L12_89051	2765	*grp-94*	XP_023283565	0	104-2509	MH138015
L2_65026	450	*grp-170*	ADX97080	4e-77	<1->450	MH138016
L12_51413	785	*grp-78*	KKF16569	1e-150	165->785	MH138017
L3_44203	706	*mthsp10*	XP_011605755	8e-61	249-548	MH138018
L1_45931	726	*grp-75*	ABF70952	7e-145	<1->726	MH138019

[a] Gene identity determined through BLAST-X searches: *leap2*:liver-expressed antimicrobial peptide 2; *lyz*: lysozyme C; *defb*: beta-defensin; *igmb*: IgM membrane-bound; *a2m*: alpha-2 macroglobulin; *lgals8x1*: galectin-8-like isoform X1; *ucp1*: mitochondrial respiratory uncoupling protein 1; *sirt1*: sirtuin 1; *sirt5*: sirtuin 5; *cat*: catalase; *mn-sod*: superoxide dismutase Mn; *gpx1*: glutathione peroxidase 1; *gpx4*: glutathione peroxidase 4; *gr*: glutathione reductase; *prdx1*: peroxiredoxin 1; *prdx4*: peroxiredoxin 4; *prdx5*: peroxiredoxin 5; *prdx6*: peroxiredoxin 6; *grp-94*: glucose-regulated protein 94; *grp-170*: glucose-regulated protein 170; *grp-78*: glucose-regulated protein 78; *mthsp10*: mitochondrial Hsp10; *grp-75*: mitochondrial Hsp70 mortalin. [b] Best BLAST-X protein sequence match (lowest E value). [c] Expectation value. [d] Codifying sequence.

Table S6 – Two-way ANOVA of gene expression levels in the head kidney of seabass fed the experimental diets and infected with Phdp or placebo. Data expressed as mean ± SD (N=20 fish per group). All data are normalized to the expression level of *cd247* of fish fed control diet and non-infected, with an arbitrarily assigned value of 1.

	**Dietary treatments**								***p* value**		
	**Control**				***Gracilaria* sp.**						
	**Placebo**		**Infected**		**Placebo**		**Infected**				
**Genes**	**Mean**	**SD**	**Mean**	**SD**	**Mean**	**SD**	**Mean**	**SD**	**Diet**	**Infection**	**D x I**
a2m	0.76	0.31	1.59	0.64	0.14	0.05	0.11	0.03	**0.008**	0.940	0.938
ackr4	1.87	0.87	0.16	0.01	0.28	0.04	0.35	0.05	0.068	0.147	**0.018**
defb	0.03	0.02	0.00	0.00	0.00	0.00	0.00	0.00	**0.006**	0.275	0.285
ccr3	1.48	0.25	1.00	0.10	1.62	0.27	1.38	0.21	0.310	0.127	0.614
ccr9	1.39	0.29	0.84	0.07	2.01	0.44	2.48	0.59	**0.010**	0.910	0.223
c3	0.54	0.24	1.13	0.43	0.16	0.07	0.11	0.04	0.881	0.955	0.968
g8x1	4.22	0.60	2.84	0.18	2.74	0.31	3.10	0.42	0.134	0.211	**0.036**
igmb	25.59	8.79	10.68	1.24	32.83	6.57	29.83	8.70	0.066	0.205	0.397
igms	67.49	17.86	126.166	19.26	93.75	19.41	290.790	55.32	**0.010**	**< 0.001**	0.077
irf8	6.64	1.48	4.96	0.31	6.59	0.95	8.79	1.51	0.179	0.995	0.074
il1b	0.20	0.10	0.26	0.07	0.06	0.02	0.05	0.01	**0.002**	0.214	0.102
il10	0.17	0.03	0.17	0.02	0.15	0.02	0.22	0.03	0.690	0.163	0.192
il20	0.13	0.03	0.06	0.01	0.06	0.01	0.07	0.01	0.093	0.693	0.189
il34	0.48	0.09	0.22	0.02	0.73	0.08	0.58	0.07	**< 0.001**	**0.006**	0.400
il8	0.56	0.13	0.39	0.08	0.48	0.08	0.77	0.21	0.554	0.545	0.696
leap2	0.02	0.00	0.01	0.00	0.01	0.00	0.01	0.00	0.392	**0.010**	**< 0.001**
lyz	3.61	1.17	1.26	0.52	0.62	0.26	0.32	0.07	0.638	0.374	0.395
csf1r	3.59	0.78	3.42	0.35	3.40	0.63	6.06	1.24	0.144	0.137	0.093
mif	22.23	3.20	21.74	1.76	8.99	1.09	14.35	2.12	**< 0.001**	0.266	0.185
mmd	2.45	0.53	1.38	0.12	1.14	0.12	1.39	0.13	0.076	0.805	**0.007**
cd33	2.59	0.62	2.57	0.22	5.16	0.96	5.25	1.20	**0.038**	0.801	0.213
myd88	6.72	0.70	7.60	0.32	5.72	0.39	6.54	0.42	**0.036**	0.078	0.953
nfkb2	8.93	1.09	6.84	0.40	7.94	0.85	9.61	1.44	0.897	0.578	0.340
crp	41.80	8.13	51.20	4.32	37.11	4.41	41.81	3.77	0.191	0.190	0.659
sap	0.05	0.01	0.06	0.01	0.08	0.01	0.05	0.01	0.100	0.372	**0.022**
cd247	1.14	0.27	0.82	0.06	0.97	0.19	1.80	0.41	0.121	0.315	**0.030**
cd8b	0.79	0.17	0.33	0.05	0.57	0.07	1.20	0.33	0.875	**0.011**	0.737
trf	58.23	35.41	6.90	2.72	1.02	0.45	0.63	0.13	0.689	0.147	0.246
tnfα	0.34	0.06	0.20	0.01	0.22	0.03	0.31	0.05	0.953	0.930	**0.041**

Significant differences ($p<0.05$) in bold.

Table S7– Two-way ANOVA of gene expression levels in the spleen of seabass fed the experimental diets and infected with Phdp or placebo. Data are expressed as mean ± SD (N=20 fish per group). All data are normalized to the expression level of *ackr4* of fish fed control diet and non-infected, with an arbitrarily assigned value of 1.

	**Dietary treatments**								***p* value**		
	**Control**				***Gracilaria* sp.**						
	**Placebo**		**Infected**		**Placebo**		**Infected**				
**Genes**	**Mean**	**SD**	**Mean**	**SD**	**Mean**	**SD**	**Mean**	**SD**	**Diet**	**Infection**	**D x I**
a2m	0.50	0.17	0.26	0.10	0.47	0.21	0.19	0.05	0.179	0.692	0.690
ackr4	1.06	0.11	0.77	0.07	1.21	0.09	1.07	0.10	**0.032**	**0.037**	0.996
defb	0.01	0.00	0.02	0.01	0.02	0.01	0.02	0.00	0.146	0.253	0.094
ccr3	0.49	0.08	0.59	0.09	1.04	0.14	1.04	0.17	**0.014**	0.172	0.684
ccr9	1.28	0.10	0.75	0.08	1.31	0.14	1.23	0.11	0.073	0.057	0.074
c3	0.44	0.14	0.27	0.12	0.60	0.21	0.17	0.06	0.189	0.449	0.528
g8x1	2.21	0.20	1.81	0.16	1.76	0.10	1.93	0.12	0.525	0.091	0.136
igmb	7.52	1.42	3.81	1.04	10.33	1.74	9.04	1.48	**0.026**	0.171	0.124
igms	29.65	2.70	68.88	20.17	58.48	11.75	75.037	11.40	0.078	0.076	0.234
irf8	8.36	0.63	6.99	0.65	7.53	0.51	7.58	0.32	0.396	0.376	0.368
il1b	0.06	0.02	0.12	0.03	0.07	0.04	0.02	0.01	**< 0.001**	0.708	0.059
il10	0.34	0.06	0.22	0.03	0.38	0.11	0.24	0.03	0.857	0.927	0.783
il20	0.15	0.02	0.10	0.02	0.17	0.03	0.14	0.02	0.262	0.188	0.241
il34	3.62	0.38	1.90	0.20	3.53	0.40	2.68	0.29	0.440	**0.001**	0.175
il8	0.41	0.04	0.33	0.06	0.27	0.04	0.39	0.07	0.295	0.248	0.305
leap2	0.02	0.01	0.01	0.00	0.04	0.01	0.02	0.00	**0.015**	**0.010**	**0.006**
lyz	1.20	0.43	0.29	0.18	2.08	0.86	0.46	0.15	0.624	**0.023**	0.559
csf1r	9.97	0.98	7.66	0.60	6.68	0.40	10.17	0.91	0.568	0.305	**0.038**
mif	4.81	0.41	4.58	0.42	4.66	0.37	4.81	0.91	0.416	0.189	0.885
mmd	0.56	0.04	0.63	0.06	0.50	0.04	0.54	0.03	0.261	0.786	0.706
cd33	17.78	1.40	16.63	1.12	16.11	1.21	13.90	1.35	0.185	0.082	0.299
myd88	3.52	0.36	3.72	0.23	3.21	0.25	3.02	0.18	0.178	0.729	0.606
nfkb2	5.80	0.35	4.41	0.38	6.01	0.62	5.12	0.29	0.292	**0.004**	0.111
crp	16.77	1.51	19.42	1.74	11.75	1.03	16.80	2.62	**0.005**	0.303	0.958
sap	0.01	0.00	0.01	0.00	0.02	0.01	0.02	0.01	0.816	0.590	0.514
cd247	2.09	0.24	1.22	0.14	3.11	0.79	2.01	0.18	0.908	0.795	**0.017**
cd8b	0.44	0.03	0.29	0.04	0.68	0.08	0.57	0.06	**< 0.001**	**0.009**	0.936
trf	3.81	1.57	1.90	0.72	2.85	0.93	2.00	0.57	0.768	0.862	0.542
tnfα	0.28	0.03	0.22	0.02	0.29	0.04	0.24	0.03	0.844	**0.012**	0.752

Significant differences (p<0.05) in bold.

Table S8– Two-way ANOVA of gene expression levels on the liver of seabass fed the experimental diets and infected with Phdp or placebo. Data are expressed as mean ± SD (N=10 fish per group). All data are normalized to the expression level of *grp-78* of fish fed control diet and non-infected, with an arbitrarily assigned value of 1.

	Dietary treatments								*p* value		
	Control				*Gracilaria* sp.						
	Placebo		Infected		Placebo		Infected				
**Genes**	**Mean**	**SD**	**Mean**	**SD**	**Mean**	**SD**	**Mean**	**SD**	**Diet**	**Infection**	**D x I**
cs	0.70	0.06	0.67	0.05	0.57	0.08	0.58	0.03	**0.029**	0.810	0.531
coxi	94.74	15.01	72.11	9.33	65.72	12.33	45.88	6.57	**0.012**	0.103	0.940
ucp1	2.01	0.33	1.02	0.13	2.70	0.25	2.25	0.20	**< 0.001**	**0.011**	0.160
sirt1	0.04	0.00	0.04	0.00	0.02	0.00	0.03	0.00	**< 0.001**	0.053	0.355
sirt5	0.27	0.02	0.26	0.02	0.21	0.02	0.17	0.01	**< 0.001**	0.162	0.426
cat	8.21	0.39	6.95	0.92	9.05	0.88	6.18	0.31	0.951	**0.004**	0.244
mn-sod / sod2	0.69	0.08	0.40	0.05	0.89	0.12	0.535	0.05	**0.018**	**< 0.001**	0.858
gpx1	0.51	0.05	0.51	0.06	0.45	0.06	0.36	0.03	0.051	0.413	0.443
gpx4	26.36	2.56	22.17	2.15	18.88	1.19	23.23	1.67	0.111	0.969	**0.036**
gr	0.16	0.02	0.19	0.02	0.12	0.01	0.10	0.01	**0.001**	0.551	0.200
prdx1	1.90	0.62	1.55	0.22	0.87	0.09	0.52	0.08	**< 0.001**	**0.010**	**0.004**
prdx4	2.03	0.22	1.25	0.15	1.84	0.10	0.91	0.07	0.093	**< 0.001**	0.246
prdx5	0.14	0.01	0.20	0.03	0.22	0.02	0.12	0.01	0.671	0.281	**< 0.001**
prdx6	5.54	0.52	4.07	0.36	5.76	0.46	5.19	0.59	0.178	**0.043**	0.360
grp-94	2.37	0.39	3.44	0.52	1.36	0.19	0.59	0.10	**< 0.001**	0.705	**0.006**
grp-170	0.57	0.12	1.45	0.21	0.19	0.04	0.19	0.08	**< 0.001**	0.079	**0.009**
grp-78	1.29	0.26	3.22	0.60	0.33	0.07	0.23	0.04	**< 0.001**	0.092	**0.005**
mthsp10	0.47	0.06	0.65	0.11	0.50	0.08	0.30	0.04	**0.014**	0.529	**0.012**
grp-75	0.63	0.09	0.86	0.16	0.35	0.04	0.35	0.05	**< 0.001**	0.464	0.396

Significant differences ($p<0.05$) in bold.

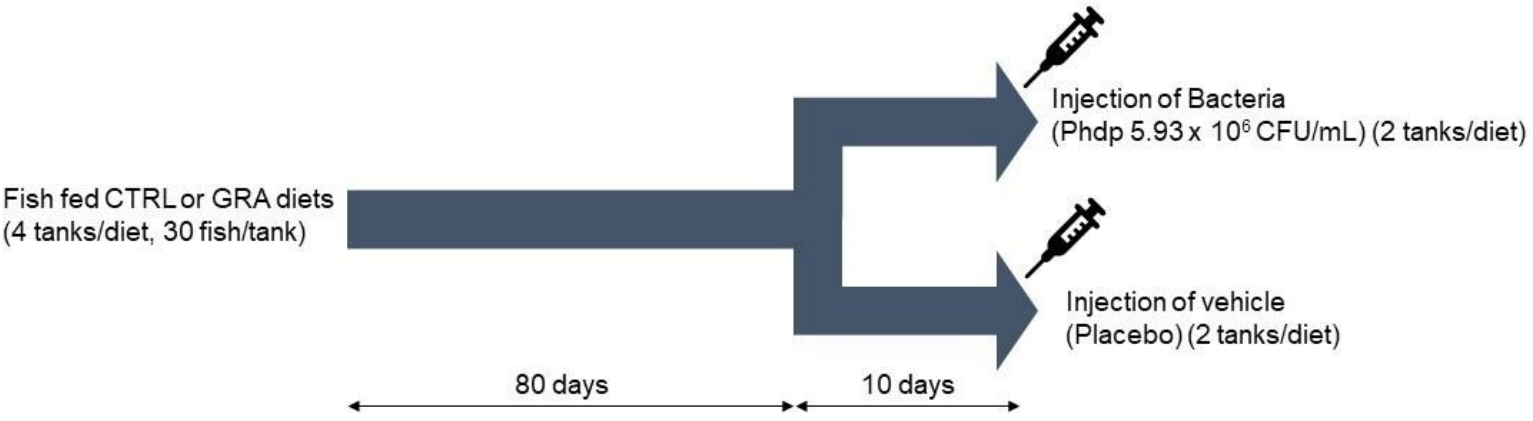

Endpoints (Day 90)	Experimental Unit; N
Weight	Tank; 2
Mortality	Tank; 2
Plasma Metabolites	Fish; 16
Immune Parameters	Fish; 16
Liver Enzymes	Fish; 16
Gene expression	Fish; 20

Figure S1 – Experimental methodology.

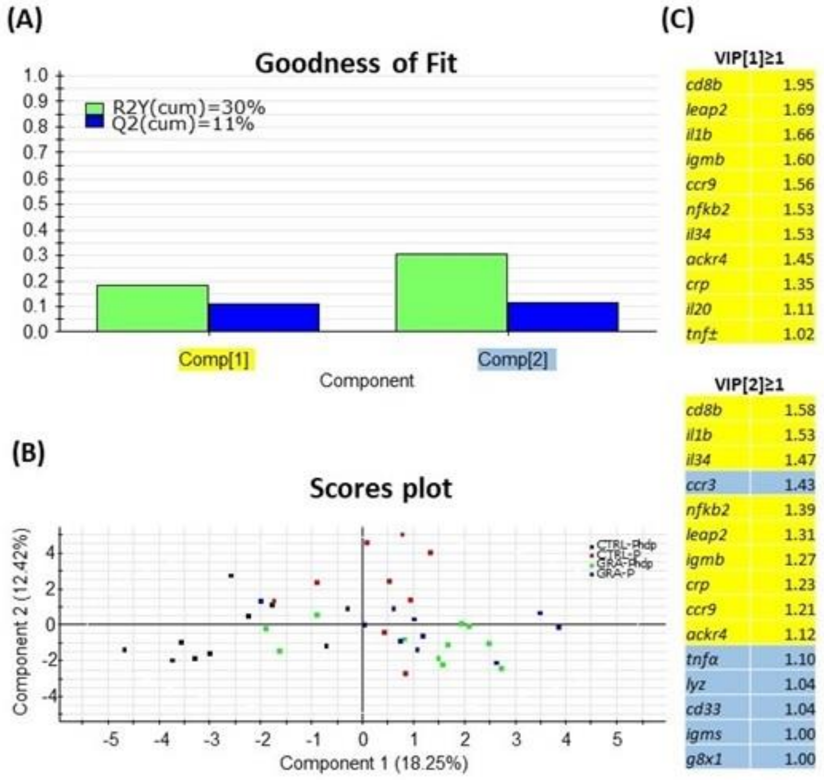

Figure S2 – Discriminant analysis (PLS-DA) of spleen molecular signatures of seabass, altered by dietary *Gracilaria* sp. supplementation and Phdp infection.
